# Andrographolide: A New Plant-Derived Antineoplastic Entity on Horizon

**DOI:** 10.1093/ecam/nep135

**Published:** 2011-02-10

**Authors:** Astha Varma, Harish Padh, Neeta Shrivastava

**Affiliations:** B. V. Patel Pharmaceutical Education & Research Development (PERD) Centre, Sarkhej—Gandhinagar Highway, Thaltej, Ahmedabad 380054, Gujarat, India

## Abstract

Plant-derived natural products occupy an important position in the area of cancer chemotherapy. Molecules such as vincristine, vinblastine, paclitaxel, camptothecin derivatives, epipodophyllotoxin, and so forth, are invaluable contributions of nature to modern medicine. However, the quest to find out novel therapeutic compounds for cancer treatment and management is a never-ending venture; and diverse plant species are persistently being studied for identification of prospective anticancer agents. In this regard, *Andrographis paniculata* Nees, a well-known plant of Indian and Chinese traditional system of medicines, has drawn attention of researchers in recent times. Andrographolide, the principal bioactive chemical constituent of the plant has shown credible anticancer potential in various investigations around the globe. *In vitro* studies demonstrate the capability of the compound of inducing cell-cycle arrest and apoptosis in a variety of cancer cells at different concentrations. Andrographolide also shows potent immunomodulatory and anti-angiogenic activities in tumorous tissues. Synthetic analogues of the compound have also been created and analyzed, which have also shown similar activities. Although it is too early to predict its future in cancer chemotherapy, the prologue strongly recommends further research on this molecule to assess its potential as a prospective anticancer agent.

## 1. Introduction

### 1.1. The Anti-Neoplastic Phyto-Pharmacophores

The era of chemotherapy began in 1940s with the first use of nitrogen mustards and antifolate drugs [1]. Thereafter, cancer drug discovery and development have been the major research endeavor around the globe. The quest to find new therapeutic candidate compounds from natural biodiversity, particularly plants, has been the prime interest amongst researchers. The search for anti-cancer agents from plant sources started in earnest in 1950s with the discovery and development of the vinca alkaloids, vinblastine (velban) and vincristine (oncovin); and the isolation of cytotoxic podophyllotoxins [2]. Vinca alkaloids (antimitotics, which form tubulin-alkaloid complexes and distort the microtubule assembly of cancer cells) are generally used in combination therapy with synthetic molecules [3–5]. Most recent semi-synthetic analogues of these agents are vinorelbine (navelbine) and vindesine (eldisine). Another class of potent plant-derived anti-cancer agents consists of topoisomerase II inhibitors. Etoposide (Vepesid) and teniposide (Vumon) are semi-synthetic derivatives of epipodophyllotoxin (isomer of podophyllotoxin) derived from *Podophyllum* spp. (*Podophyllum peltatum* Linnaeus and *Podophyllum emodi* Wallich). Most recent additions to the armamentarium of plant-derived anti-cancer agents are taxanes and camptothecins. Paclitaxel (commonly known as Taxol) and the related semi-synthetic docetaxel (Taxotere) are currently being used in a large number of cancer treatments [6]. Though discovered quite early in drug discovery process [7], the development of these molecules as clinically active agents required about 20–30 years of dedicated research. Other molecules in clinical use are homoharringtonine, isolated from the Chinese tree, *Cephalotaxus harringtonia* var. *drupacea* (Sieb and Zucc.) (family, Cephalotaxaceae) and elliptinium, a derivative of ellipticine, isolated from species of several genera of the Apocynaceae family, including *Bleekeria vitensis* A.C. Sm., a Fijian medicinal plant with reputed anti-cancer properties [2].

In addition to these molecules in clinical use, a few very promising compounds with strong anti-cancer potential are currently undergoing clinical trials. These include flavopiridol, combretastatins, 4-ipomeanol, colchicines, genistein, lapachol, curcumin, and so forth. Flavopiridol is a synthetic flavone structurally based on alkaloid rohitukine found in *Amoora rohituka* and *Dysoxylum binectariferum* (Maliaceae). It is the most interesting plant-based compound in development as it represents the first cyclin dependent kinase (cdk) inhibitor to enter the clinic [4]. The combretastatins (isolated from South African bush willow *Combretum caffrum* (Eckl. & Zeyh.) Kuntze (family, Combretaceae)) are a family of stilbenes, which act as anti-angiogenic agents, causing vascular shutdown in tumors and resulting in tumor necrosis [2]. The demand for these anti-cancer compounds is ever increasing as strongly reflected in the annual sales of these compounds. Camptothecin derivatives account for nearly a billion dollars annually, paclitaxel and its derivatives have sales exceeding towards two billion dollars per annum [8, 9].

It is fascinating to note that almost all the above-mentioned drugs have a very strong ethno-botanical background, which strengthens the fact that by exploring the folkloric knowledge, several new compounds may be discovered. Traditional medicinal systems have always contributed chemical entities with attractive scaffolds for drug discovery [10–12]. A recent example of this approach to discover novel anticancer dugs is the formation of a library of 531 cytotoxic natural products derived from traditional Chinese medicine (TCM) [13]. However, this is just a glimpse of what the plant biodiversity holds for us in the area of anti-cancer research. While many plant-derived molecules have shown wonders as chemotherapeutic agents, there are a large number of compounds that need to be explored as prospective anti-cancer agents. This article focuses on the anticancer potential of “andrographolide”, the major bioactive constituent of *Andrographis paniculata*, a well-known plant of Ayurveda and TCM.

### 1.2. The Plant—Andrographis Paniculata Nees

**Figure 1:**
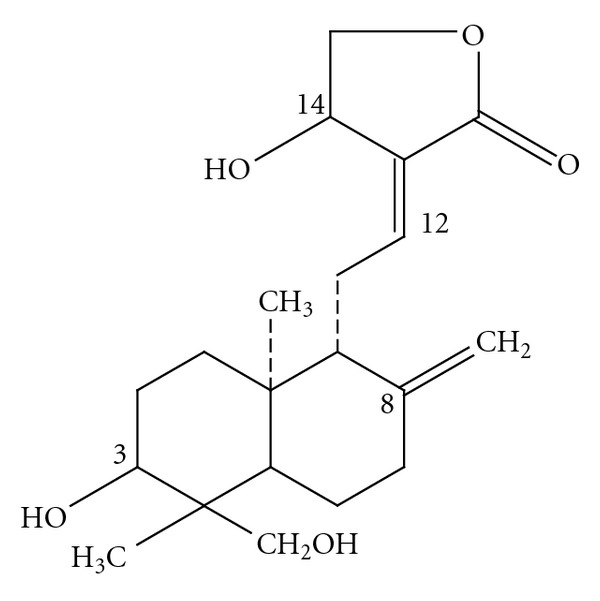
Structure of the principle phytochemical compound of *A. paniculata* Nees, andrographolide.

*Andrographis paniculata* (Burm. F.) Nees (family, Acanthaceae) grows widely in many Asian countries such as China, India, Thailand and Sri Lanka [14] and has a long history of therapeutic usage in Indian and Oriental medicine [15, 16]. The herb is official in Indian Pharmacopoeia [17] as a predominant constituent of at least 26 Ayurvedic formulations used to treat liver disorders. It is one of the herbs, which can be used to treat neoplasm as mentioned in ancient Ayurvedic literature [18]. *Andrographis paniculata* is reported as a cold property herb in TCM and is used to get rid of body heat and to expel toxins. The plant is particularly known for its extremely bitter properties (often called king of bitters) and is used traditionally as a remedy against common cold, dysentery, fever, tonsillitis, diarrhoea, liver diseases, inflammation, herpes, and so forth [19–21]. The traditional uses and pharmacological aspects of *A. paniculata* have been well-documented in an extensive review recently [22]. A number of active principles are reported from the plant, which mainly include diterpene lactones, flavonoids and polyphenols [23, 24]. However, the prime constituent andrographolide has been is mainly attributed for its therapeutic properties. Diterpenoid lactone andrographolide (C_20_H_30_O_5_) is the principle compound found in *A. paniculata*, which is mainly concentrated in leaves and can be easily isolated from the crude plant extracts as crystalline solid [25, 26]. The structure of the compound has been elucidated by X-Ray crystallographic analysis and the molecular stereochemistry, bond distances, bond angles, and so forth all were determined [27]. Chemically designated as (3-[2-[decahydro-6-hydroxy-5-(hydroxymethyl)-5, 8-adimethyl-2-methylene-1-napthalenyl] ethylidene] dihydro-4-hydroxy-2(3*H*)-furanone), andrographolide (Figure 1) exhibits extraordinarily vast range of biological activities [28–33]. In recent past, the compound is reported for its anti-tumor, anti-HIV and cardio-protective properties [15, 34–39]. However, it shows a weak anti-microbial activity against bacteria and viruses [40].

#### 1.3. Anti-Cancer Potential of Andrographolide

Most of the anti-cancer agents employed in modern medicine aim at inhibiting the proliferation of cancer cells by inducing apoptosis, necrosis, cell-cycle arrest or cell differentiation; others might involve immunomodulatory activity, by triggering body's own immune system against these cells. The compounds that inhibit multiple procancer events are of greater interest as they are more likely to inhibit a wider range of cancers under great variety of circumstances [41]. In this context, andrographolide presents a strong candidature as a therapeutic anticancer pharmacophore as it exhibits a dual property, acting both directly and indirectly on the cancer cells [42], which will be discussed in detail in this article.

##### 1.3.1. Cytotoxic Activity against Cancer Cells

Methanolic extract of *A. paniculata* has shown significant toxicity against KB (human epidermoid leukemia) and P388 (lymphocytic leukemia) cell lines [43]. Bioactivity guided chromatographic fractionation led to the isolation of pure andrographolide, which was also highly toxic to the above-mentioned cell lines. This was one of the first significant demonstrations of cytotoxic potential of andrographolide. Potent cytotoxicity in a dose dependent manner towards various kinds of cancer cell lines including drug resistant cancer cells has also been reported in another excellent work [37]. The cytotoxic property has been attributed to the ability of andrographolide to inhibit proliferation and induce apoptosis in cancer cells.

##### 1.3.2. Induction of Cell-Cycle Arrest

Various studies have demonstrated that andrographolide effectively induces cell-cycle arrest in cancer cells at G0/G1 stage [44]. A study with human acute myeloid leukemic HL-60 cells, demonstrated a 27% increase in G0/G1 phase cells and significant decrease in cells at S and G2/M phase after andrographolide treatment (12 *μ*g/ml) for 36 h [45]. Andrographolide inhibits cell-cycle progression by modulating the expression of cell-cycle related proteins. The induction of cell-cycle arrest at G0/G1 phase is mainly due to the induction of cell-cycle inhibitory proteins p16, p21, p27 associated with decreased expression of cyclin A, cyclin D, CDK4 and CDK2, required for G1 to S transition [37, 46]. Shi et al. [46] have demonstrated almost complete inhibition of human colorectal carcinoma Lovo cells as attained by andrographolide treatment (10–30 *μ*M). Here the increased levels of p21 after andrographolide treatment (3.75-fold) are of particular interest as decreased p21 expression has been associated with aggressive phenotype in many cancers. The molecular target of andrographolide that blocks the G1 stage still needs to be determined.

##### 1.3.3. Induction of Apoptosis

Andrographolide activates the extrinsic death receptor pathway (including caspase-3 and caspase-8) and induces apoptotic cell death in certain human cancer cell types [47]. In some cell types (type 1), the activation of caspase-8 is sufficient to activate the effector caspases (caspase 3/7), whereas in majority of cell types (type 2), the effector caspase activation requires amplification of signal through mitochondria. This was elucidated through another study on three different human cancer lines (including cervical, breast and hepatoma cell lines) by Zhou et al. [48], in which around 8-fold increase in the caspase 3/7 activity was observed after treatment with andrographolide (50 *μ*M for 6 h), against control [48]. The pro-apoptotic Bcl-2 family members (bid and bax) are the key mediators in relaying cell death signaling initiated by andrographolide from caspase-8 to mitochondria and then to downstream effector caspase 3, eventually leading to cytochrome *c* release and apoptotic cell death [48, 49]. A recent work demonstrates that tumor necrosis factor-*α* (TNF-*α*) related apoptosis inducing ligand (TRAIL—an important member of extrinsic apoptosis pathway) was significantly enhanced in various human cancer cell lines after treatment with andrographolide, [50]. TRAIL is an important anti-cancer agent, as it can preferentially kill cancer cells amongst normal cells and therefore is a very important molecule in cancer research [51]. Some kinds of cancer cells develop resistance towards TRAIL, which is a major constraint in TRAIL mediated apoptosis. Thus, compounds that enhance TRAIL expression or are able to re-sensitize resistant cancer cells to TRAIL induced apoptosis are extremely valuable [52, 53]. In this context andrographolide is a promising molecule as it could enhance TRAIL expression via up-regulation of death receptor (DR-4) and also re-sensitize resistant cancer cells to TRAIL-induce apoptosis [50]. Further studies in this direction might help in developing andrographolide as a sensitizer for TRAIL induced apoptosis in various kinds of tumors.

Studies have demonstrated that andrographolide is also effective in combination therapy. Andrographolide increased the apoptosis rate in multidrug resistant cancer cells, when used in combination treatment along with other anticancer agents like 5-florouracil (5-FU), adriamycin and cisplatin [54]. Andrographolide individually as well as in combination with 5-FU was assessed in treatment of human carcinoma HCC cells, where it could induce synergistic apoptosis [55]. Apart from inducing apoptosis in cancer cells, the compound is also able to induce cell differentiation in proliferating cancer cells. The myeloid leukemia (M1) cells of mouse were directed to differentiate into phagocytes following treatment with andrographolide. This particular activity is rarely found in plant-derived anti-cancer agents and thus is of particular interest [56].

##### 1.3.4. Immunostimulating Properties

*Andrographis paniculata* is known to exert a strong immunomodulatory effect as it has been observed that the alcohol extract of the plant as well as isolated andrographolide are able to induce significant stimulation of both “antigen specific” and “antigen nonspecific” types of immune responses in mice, showing effectiveness against a variety of infectious and oncogenic (cancer causing) agents [57, 58]. Factors like (TNF-*α*), interleukin-2 (IL-2), interferon-*γ* (IFN-*γ*) and natural killer (NK) cells play an important role in conferring protection against neoplastic factors. Andrographolide plays a role in regulating the production of these factors, thus acting in a circuitous manner on the cancer cells. Administration of andrographolide led to enhanced production of TNF-*α* and expression of CD markers, eventually increasing the cytotoxic activity of lymphocytes against cancer cells [37]. An increased proliferation of human peripheral blood lymphocytes (HPBLs) was observed after andrographolide treatment (1 *μ*M for 48 h), owing to enhanced IL-2 production and ultimately the immune response against cancer cells [59]. *In vivo* experiments show that antibody-dependent cellular toxicity, mitogen induced proliferation of bone marrow cells and production of Il-2 and IFN-*γ* was elevated on treatment with andrographolide in normal as well as carcinoma bearing animals [60]. Andrographolide stimulated the production of cytotoxic T lymphocytes inhibiting tumor growth in animals [61]. When administered in combination with other neutraceuticals, andrographolide caused an increase in function of NK cells and TNF-*α* thus resulting in improved clinical outcomes in patients with late stage cancers of different types [62]. Thus the compound in addition to conferring direct toxicity to cancer cells; modulates the host immune system against these cells.

##### 1.3.5. Anti-Inflammatory and Anti-Angiogenic Activity

Both *A*. *paniculata* plant extract and andrographolide are known to have an anti-inflammatory potential [63, 64]. Inflammation is considered as a critical component of tumor progression as tumor microenvironment is largely orchestrated by inflammatory cells. This has been elucidated in an excellent review [65], which also emphasizes on anti-inflammatory therapeutic approaches for cancer treatment. Shen et al. [32] suggested the prevention of production of reactive oxygen species (ROS) by andrographolide as the possible mechanism of its anti-inflammatory effect. Andrographolide treatment inhibits nuclear factor kappa B (NF-*κ*B) binding to DNA and thus reducing the expression of pro-inflammatory proteins such as cycloxygenase 2 (Cox-2) and nitric-oxide synthase (NOS) [66–69]. An important landmark in deciphering the mechanism of action of andrographolide was the finding that andrographolide reduces cysteine 62 of p50 (a major subunit of NF-kB transcription factors), thus blocking their binding to the promoters of their target genes [39, 68]. A major constraint in chemotherapy is the acquired resistance of cancer cells to various chemotherapeutic agents by activation of NF-kB that promotes cell survival [70]. Thus andrographolide as an inhibitor of NF-*κ*B, might also be used to sensitize cancer cells to overcome such kind of resistance. Andrographolide also inhibits Erk 1/2 and Akt signaling, thus restraining the chemo-tactic migration of macrophages on inflammation site [71–73]. Inhibition of Erk signaling also leads to inhibition of v-Src oncoprotein mediated transformation, which is strongly associated with cancer initiation and progression [71].

**Figure 2:**
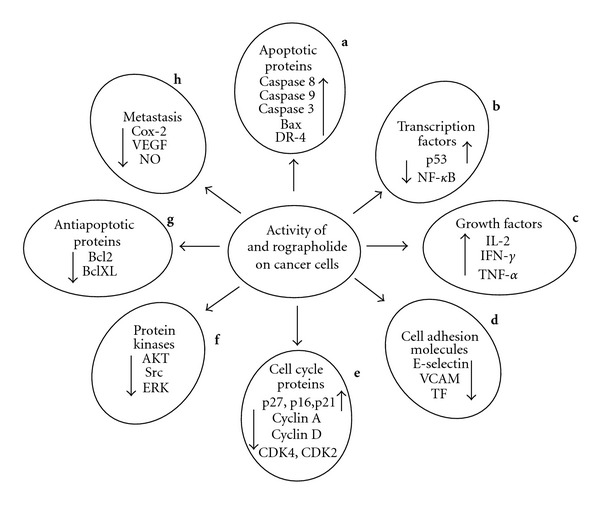
Effect of andrographolide treatment on cancer cells. Cancer is a multifaceted disease with complex processes and requires a multi-target therapeutic approach to battle it. A similar kind of action is displayed by andrographolide as it modulates various biochemical pathways of cancer cells thereby inhibiting the tumor growth. The compound exerts cytotoxic effect on various cancer cell types in a time and dose dependent manner. Factors required for tumor progression, nourishment and metastasis are down regulated, that is, cyclins A, D, Cdk2, Cdk4, NF-*κ*B, VEGF, E-selectin, VCAM, Akt, TNF, Bcl2, and so forth. On the other hand tumor suppressor elements like p53, caspases, inhibitory proteins p21, p16, p27, and so forth are up regulated as observed in various studies to investigate anti-cancer potential of andrographolide. Up regulation of death receptor 4 to facilitate TRAIL induced apoptosis is of significant interest. The cumulative effect of all these factorial events leads to inhibition of growth in cancer cells. The alphabets placed adjacent to the petals refer to the reference numbers of articles. **a** [35, 45, 47, 49, 50]; **b** [49, 50, 60, 66, 68]; **c** [60, 62]; **d** [82]; **e** [37, 46, 87]; **f** [71–73]; **g** [34, 37, 45, 48]; **h** [34, 60, 74].

Cancer cells are known to induce angiogenesis for continuous supply of nutrients to the proliferating cells. As angiogenesis is triggered in response to chronic inflammation, there is a direct relation between inflammation, carcinogenesis and angiogenesis. Therefore, owing to its excellent anti-inflammatory activity, andrographolide has been evaluated for its anti-angiogenic potential as well. As an anti-angiogenic prospect, andrographolide could successfully inhibit the tumor specific capillary sprouting without damaging the pre-existing vasculature. Andrographolide administration also down-regulated the production of various angiogenic factors like vascular endothelial growth factor (VEGF), nitric oxide (NO) and pro-inflammatory cytokines and elevated the levels of anti-angiogenic factors like IL-2 and tissue inhibitor of metalloproteinase (TIMP-1) *in vitro* as well as *in vivo* [74]. VEGF is by and large used by cancer cells as a survival factor and the inhibitory activity of andrographolide on VEGF levels has been verified [34]. A schematic diagram depicting the multitarget potential of andrographolide is presented as Figure 2.

##### 1.3.6. Chemo-Protective Potential

Andrographolide exhibits selective cytotoxicity against various cancer cells, as described earlier in the article. However, the compound has also shown a chemo-protective potential towards normal cells in a few studies. The chemo-protective potential of plant extract against chemo-toxicity including carcinogenicity has been described earlier [75]. Andrographolide in a dose dependent manner inhibited IFN-*γ* and IL-2 production in murine thymocytes induced by concanavaline A. It also prevented cell apoptosis induced by drugs like hydrocortisone [76]. The protective effect of andrographolide against cyclophosphamide induced urothelial toxicity [77] and hexachlorocyclohexane induced oxidative injury [78] has been reported. Andrographolide is effective against a large number of hepatotoxins, which might be due to its ability to activate antioxidant enzymes that catalyze reaction of oxidants in severe liver damage. The hepatoprotective effect of the compound was found comparable to that of silymarin, when tested both *in vitro* and *in vivo* [79, 80]. These studies support the fact that *A. paniculata* is used in Ayurvedic formulations to treat liver disorders. Thus, in addition to inducing cytotoxicity to cancer cells directly or indirectly, andrographolide can also prevent the cytotoxicity to normal cells induced by various agents. Chen et al. [81] have described protective function of andrographolide on human umbilical vascular endothelial cells (HUVECs) from GF deprivation-induced apoptosis via enhancement of PI3K-Akt activity. Here, andrographolide suppressed mitochondria mediated apoptosis by inhibiting cytochrome c release to cytosol. In another finding, human vascular endothelial cells were protected from adhesion of gastric cancer cells by andrographolide through blocking of E-selectin expression [82]. E-selectin is modulated by NF-*κ*B, thus demonstrating the effect of andrographolide treatment. However, andrographolide treatment can also induce expression of CYP1A subfamily of cytochrome P450 family of enzymes, which are involved in metabolism of a plethora of xenobiotics, and thus this interaction may be clinically significant [83]. Thus, further studies to determine the effect on the compound on cytochrome P450 enzymes are fairly imperative.

#### 2. Analogues of Andrographolide

In addition to the naturally found andrographolide, researchers have been successful in synthesizing andrographolide derivatives that have also displayed anti-cancer activities [84]. By parallel solution phase synthesis, Mang et al. [85] have generated a 360 membered natural product library starting from andrographolide. Naturally occurring andrographolide (Figure 1) contains: (i) an *α*-alkylidene-c-butyrolactone moiety, (ii) two double bonds *δ* ^8(17)^ and *δ* ^12(13)^ and (iii) three hydroxyls at C-3, C-19 and C-14. Of the three hydroxyls, the one at C-14 is allylic, while others at C-3 and C-19 are secondary and primary, respectively [86]. The intact *γ*-butyrolactone ring, the double bonds between C-12 and C-13, C-8 and C-17 and hydroxyl group at C-14, are primarily responsible for the cytotoxic activity of the compound. Modifications made to this primary skeleton of andrographolide may improve its anti-tumor activity [36, 87]. Three analogues (3,19-isopropylideneandrographolide; 14-acetyl-3, 19-isopropylideneandrographolide and 14-acetylandrographolide) were synthesized by Jada et al. [36] employing andrographolide as the starting material, of which 14-acetylandrographolide was significantly more potent against many cancer cell lines when compared with the parent compound. However, the mechanism of inducing cell-cycle arrest was different from andrographolide. This research group has successfully developed new benzylidene derivatives of andrographolide [3,19-(2-bromobenzylidene) andrographolide and 3,19-(3-chloro-4-fluorobenzylidene) andrographolide], which are more cytotoxic and potent than andrographolide [88]. Another semi-synthetic analogue of andrographolide, DRF 3188 was found to have a better anti-cancer activity on the cell-cycle of MCF-7 breast cancer line by a similar mechanism as andrographolide [87]. The effect was almost comparable for both compounds, *in vitro* as well as *in vivo*. Novel family of potent and specific *α*-glucosidase inhibitors has been synthesized by using andrographolide as the parent molecule [86]. These compounds have the potential to be developed as antitumor agents. These results are supported by a different finding, where it was demonstrated that the succinoyl ester of andrographolide significantly inhibited proprotein convertases and thus displayed potent antiviral activities against HIV-1 and HIV-2 [89]. These results emphasize that some of the derivatives of andrographolide might be much more potent that the parent compound itself and call for a dedicated line of investigations to prove their potential.

#### 3. Current State of Affairs and Future Directions

There has been a significant rise in the number of studies deciphering various aspects of anti-neoplastic activity of andrographolide around the globe. However, most of the data is based on *in vitro* cellular toxicity assays. Concrete information based on mouse models of cancer is lacking and there is a dearth of clinical evidence. Although andrographolide has been subjected to clinical trials for treatment against HIV and acute upper respiratory tract infection [38, 90], clinical data regarding its anti-cancer activity is still awaited. More studies on the pharmacokinetic properties of the compound need to be performed. Therapeutic efficacy of a drug is reflected by its bioavailability and poor solubility of andrographolide in water affects its bioavailability. Recently an inclusion technique has been developed to modify its physical and chemical properties so as to increase its bioavailability as well as prevent its hydrolysis in neutral and alkaline environment of gastrointestinal tract [91]. However, when taken in the form of an extract (Kan Jang), andrographolide is readily absorbed in blood (maximum plasma concentrations reached after 1.5–2 hr of oral administration) [92]. It is suggested that P-glycoprotein participates in the intestinal absorption of andrographolide [93]. The available information on the metabolism of the compound reflects that metabolic fate of andrographolide in humans after oral administration might involve a sulphonate reaction at C-12 [94]. Structural illucidation of metabolites after oral administration of andrographolide have shown sulphate compounds and sulphonic acid adducts [95].

The available information is mostly in bits and pieces, which needs to be compiled and assessed for carrying out more appropriate studies towards establishing andrographolide as a prospective anti-cancer agent. The molecule has shown a broad-range anti-proliferative activity on a variety cancer cell lines including breast cancer, colon cancer, hepatoma, cervical cancer, leukemia, prostrate cancer and many more. However it was found most effective against colon cancer cell lines [36, 37, 44, 46, 59], followed by prostrate cancer and breast cancer. Amongst various breast cancer cell lines also, the MCF-7 cell lines were found most sensitive. As the colorectal and colon cancer cells are observed to be more sensitive towards andrographolide treatment, it would be worthy to conduct more studies on colon cancer using andrographolide as the prospective drug. The precise mechanism of action of the compound also needs to be decoded, which is possible with the current technological advances in our hands.

#### 4. Conclusion

The hunt for alternative and complementary medicine is an ongoing process in the area of cancer research, where *A. paniculata* Nees, a renowned plant in South-Asian traditional medicine has recently attracted much attention owing to its anti-cancer properties. The principle phytochemical constituent of the plant, andrographolide, has shown significant anti-neoplastic and immunomodulatory activities in a number of studies performed in recent times. It is a well-established fact that an integrated approach is needed to manage cancer and a compound or a group of compounds that can influence multiple biochemical pathways related to tumorigenesis are of prime interest in cancer chemotherapy. With reference to Ayurveda, medicines work in synergy to nourish body as a whole and thus several organ systems are affected at a time. *Andrographis paniculata* is cited in Ayurveda as a plant with anti-cancer properties and it is easy to presume the action of andrographolide on similar principles. The compound is able to induce a G0/G1 cell-cycle arrest in various kinds of cancer cells, activate the death receptor pathways, induce TRAIL mediated apoptosis, activate p53 via enhanced phosphorylation and cause inhibition of NF-*κ*B transcriptional factors and various angiogenic factors. It also exerts strong immunomodulatory effects against cancer cells in addition to its cytotoxic effects; a property which is similar to other anticancer agents including doxorubicin, mitomycin, cisplatin, and so forth. Apart from acting on various pathways to obliterate cancer cells; it also exerts a protective effect on normal cells saving them from induced toxicity, in comparison to the contrary effect against cancer cells. These characteristics make it an interesting molecule for further research. In addition to the naturally found andrographolide, semi-synthetic analogues of the compound have been synthesized, some of which have also shown strong anti-cancer properties.

However, a lot of investigation needs to be done before establishing it as a prospective chemotherapeutic agent. For instance, the exact mechanism of action of andrographolide has yet to be determined and toxicity of the compound to higher animals and humans needs to be established. It should be remembered that a number of naturally derived agents have entered the clinical trials and terminated due to lack of efficacy and largely due to unacceptable toxicity. Traditionally, the screening of compounds by performing *in vitro* bioassays on a number of cancer cell lines has led to the selection of promising compounds that are headed to pre-clinical and clinical studies. On that basis, andrographolide and its semi-synthetic derivatives are interesting prospects, which can also provide a lead for novel anti-cancer drug synthesis. Summing up, andrographolide puts forward a strong candidature as a prospective plant-derived anti-cancer entity that needs to be thoroughly investigated for its anti-cancer potential.

#### Funding

Council of Scientific and Industrial Research, Government of India and Industrial Commissionerate Gujarat, Government of Gujarat.
